# Best evidence summary and nursing decision pathway for the nursing management of drainage-associated open surgical wounds following minimally invasive treatment for severe acute pancreatitis

**DOI:** 10.1186/s12912-026-05061-5

**Published:** 2026-07-17

**Authors:** Chenyang Ai, Yuhui Tan, Zhiqi Chen, Yan Zhang, Xiuyu Liu, Jinting Li

**Affiliations:** 1 Tianjin Nankai Hospital, Tianjin Medical University, Tianjin, China; 2 Tianjin Medical University, Tianjin, China; 3 Tianjin University of Traditional Chinese Medicine, Tianjin, China

**Keywords:** Evidence-based nursing, Severe acute pancreatitis, Minimally invasive surgery, Open surgical wound, Nursing management

## Abstract

**Background:**

This study identified, assessed, and integrated the best available evidence concerning the nursing management of drainage-associated open surgical wounds following minimally invasive treatment for severe acute pancreatitis, so as to develop nursing decision pathways and practical implementation tools for clinical practice.

**Method:**

This study comprised a best evidence summary followed by translation of the synthesised evidence into a nursing decision pathway and five supporting tools. According to the 6 S evidence resource model, a systematic search was conducted for evidence related to the assessment, prevention, infection recognition, exudate management, dressing selection, drainage tube exit-site care, health education and follow-up of drainage-associated open surgical wounds after minimally invasive treatment for severe acute pancreatitis. The types of evidence included clinical decisions, clinical practice guidelines, expert consensus statements, evidence summaries, best practice recommendations, clinical pathways, systematic reviews and meta-analyses. The publication date of eligible evidence sources ranged from database inception to 30 September 2025. Methodological quality was appraised using AGREE II, AMSTAR 2 and the corresponding JBI critical appraisal tools. Directness was assessed separately by comparing each source with the target population and wound context.

**Results:**

Following evidence retrieval, screening and quality appraisal, 11 evidence sources met the inclusion criteria, including two clinical practice guidelines, one national health industry standard, two best-practice recommendation documents, three systematic reviews or meta-analyses, one international consensus document, one practice-oriented clinical framework and one local clinical pathway/practice tool. Thirty-five evidence statements were synthesised across the domains of multidisciplinary collaboration, wound assessment, infection recognition, exudate management, periwound skin protection, dressing selection, drainage tube exit-site care, health education, follow-up and referral. Based on the synthesised evidence, a nursing decision pathway and five practical tools were developed, including a wound assessment record form, an infection grading and management card, a dressing selection matrix, a drainage tube exit-site care checklist, and a health education and follow-up plan.

**Conclusion:**

This study translated dispersed wound-care evidence into an operational nursing decision pathway and five supporting tools for drainage-associated open surgical wounds following minimally invasive treatment for severe acute pancreatitis. The pathway may support more standardised and consistent nursing decision-making; however, because the evidence was largely indirect and partly practice-oriented, local adaptation and prospective clinical validation are required before routine implementation.

**Registration:**

ES20259461.

## Introduction

Acute pancreatitis is a common gastrointestinal emergency with a highly variable clinical course. Although most patients recover with supportive treatment, approximately 20%–30% develop moderately severe or severe acute pancreatitis, which is associated with organ failure, pancreatic or peripancreatic necrosis, prolonged hospitalisation and substantial treatment burden [1]. Infection of pancreatic necrosis is one of the most serious complications of severe acute pancreatitis, and mortality may reach approximately 30% when infected necrosis occurs [1]. Current management has shifted towards a multidisciplinary step-up strategy, in which percutaneous or endoscopic drainage is usually considered as the initial intervention for infected or symptomatic necrotic collections, while minimally invasive necrosectomy may be required for more extensive or refractory collections [2].

For patients undergoing minimally invasive surgical or drainage-based procedures for severe acute pancreatitis, effective source control often requires drainage catheters or surgical access channels to remove infected fluid, necrotic tissue and lavage effluent [3]. Drainage-associated open surgical wounds refer to postoperative wounds or procedure-related access sites that remain open or become separated and are repeatedly exposed to drainage fluid, lavage effluent or peritubular leakage, thereby requiring ongoing wound care and, in many cases, healing by secondary intention. These drainage tubes and access sites may become persistent sources of wound-care complexity rather than simple postoperative incision sites [4]. In clinical practice, continuous drainage, leakage of lavage fluid, tube traction, repeated dressing changes and local contamination may expose the wound edges and surrounding skin to a moist, highly exudative and irritant environment. Inadequate control of wound fluid may expose the surrounding skin to sustained moisture and irritant contact, especially in situations where drainage output overwhelms dressing absorbency or frequent dressing removal increases the risk of adhesive-related skin injury [5]. When wound edges cannot be approximated, or when the wound is intentionally left open because of contamination, tissue oedema or tissue viability concerns, healing occurs by secondary intention [6, 7]. Wounds managed by secondary intention commonly involve extended healing trajectories, increased infection risk and complex care requirements; both nurses and surgeons have described uncertainty in treatment selection, infection control, dressing choice and follow-up [8]. In patients with severe acute pancreatitis, these wound-care challenges are further complicated by systemic inflammation, nutritional compromise, pain, multiple drainage tubes and the risk of ongoing intra-abdominal infection.

Drainage-associated open surgical wounds following minimally invasive treatment for severe acute pancreatitis therefore require more than routine postoperative incision observation. Nursing management needs to integrate structured wound assessment, early recognition of local or spreading infection, exudate control, periwound skin protection, prevention of moisture-associated skin damage, drainage tube securement, pain management, patient education and timely referral [9–11]. International wound infection guidance emphasises that accurate and timely identification of wound infection is critical for effective wound management and provides a wound infection continuum to guide clinical judgement and escalation decisions [12]. Best practice recommendations for moisture-associated skin damage also highlight the need for structured, skin-focused prevention and management plans to protect skin integrity in patients exposed to wound exudate and other irritant fluids [5]. However, current recommendations are dispersed across surgical site infection prevention, wound infection management, moisture-associated skin damage and secondary intention wound care [5, 6, 13], and few directly address the combined challenges of open wounds, persistent drainage, leakage, periwound skin damage and infection risk in patients with severe acute pancreatitis. Therefore, this study aimed to systematically search, appraise and synthesise the best available evidence for the nursing management of drainage-associated open surgical wounds following minimally invasive treatment for severe acute pancreatitis, and to develop a nursing decision pathway and practical implementation tools to support standardised wound assessment, infection recognition, wound care decision-making and follow-up management.

## Methods/methodology

This study was conducted as a best evidence summary. The evidence-summary process included problem formulation, evidence retrieval, source screening, methodological quality appraisal, evidence extraction, grading and synthesis [14]. The synthesised evidence was then translated into a nursing decision pathway and five supporting tools. The clinical effectiveness of the pathway and tools was not evaluated in the present study.

### Problem establishment

Following the PIPOST framework [15], the clinical question was specified in terms of population, intervention, professionals, outcomes, setting and evidence type. The population (P) of interest was adult patients with severe acute pancreatitis who had drainage-associated open surgical wounds, or a risk of such wounds, after minimally invasive surgical or drainage-based procedures. In this study, drainage-associated open surgical wounds were operationally defined as postoperative wounds or procedure-related access-site wounds that were intentionally left open to heal by secondary intention or had partially or completely separated after the procedure and were continuously or intermittently exposed to drainage fluid, lavage effluent or peritubal leakage. Wounds intentionally left open were interpreted in accordance with the concept of healing by secondary intention [6], whereas partial or complete separation of the wound edges was interpreted as surgical wound dehiscence [11]. The definition included wounds requiring repeated assessment, dressing care, exudate management or periwound skin protection. Uncomplicated closed incisions and intact drain exit sites without persistent leakage, wound-edge separation or surrounding skin injury were excluded. The intervention (I) referred to nursing management strategies, including wound assessment, infection recognition, exudate control, periwound skin protection, dressing selection, drainage tube exit-site care, health education and follow-up. The professionals (P) applying the evidence included nurses, physicians, wound/ostomy therapists and clinical managers involved in hepatobiliary and pancreatic surgical care. The outcomes (O) focused on wound infection, pain, exudate control, periwound skin integrity and wound healing trajectory. The setting (S) was inpatient hepatobiliary and pancreatic surgery departments. The type of evidence (T) encompassed clinical decisions, clinical practice guidelines, expert consensus statements, evidence summaries, best-practice recommendations, clinical pathwayor practice tools, systematic reviews and meta-analyses. Because systematic reviews and meta-analyses were included as one type of evidence source, a PICO strategy was additionally applied when judging the eligibility of systematic reviews and meta-analyses. Thus, PIPOST was used to define the overall scope of this evidence summary, whereas PICO was used as a supplementary framework for screening systematic review evidence.

### Literature retrieval method

Guided by the 6 S hierarchy of evidence sources [16], the retrieval process was conducted sequentially across pre-appraised evidence resources, guideline repositories, professional websites and bibliographic databases. Search keywords were developed in both Chinese and English and included “severe acute pancreatitis/acute necrotising pancreatitis/pancreatic necrosis/infected pancreatic necrosis”, “minimally invasive surgery/minimally invasive necrosectomy/percutaneous drainage/peritoneal lavage/drainage tube”, “open surgical wound/surgical wound healing by secondary intention/drainage-associated wound/drain exit-site wound/surgical site infection/wound infection”, and “nursing management/wound care/exudate management/dressing/periwound skin protection/moisture-associated skin damage/health education/follow-up”. The evidence-type search terms included “guideline”, “clinical practice guideline”, “clinical decision”, “evidence summary”, “best practice”, “recommendation”, “clinical pathway”, “practice tool”, “expert consensus”, “systematic review” and “meta-analysis”. Database-specific search strategies were adapted according to the characteristics of each evidence source; procedure-related terms were used where they improved retrieval sensitivity and were further applied during eligibility screening.

**Fig. 1:**
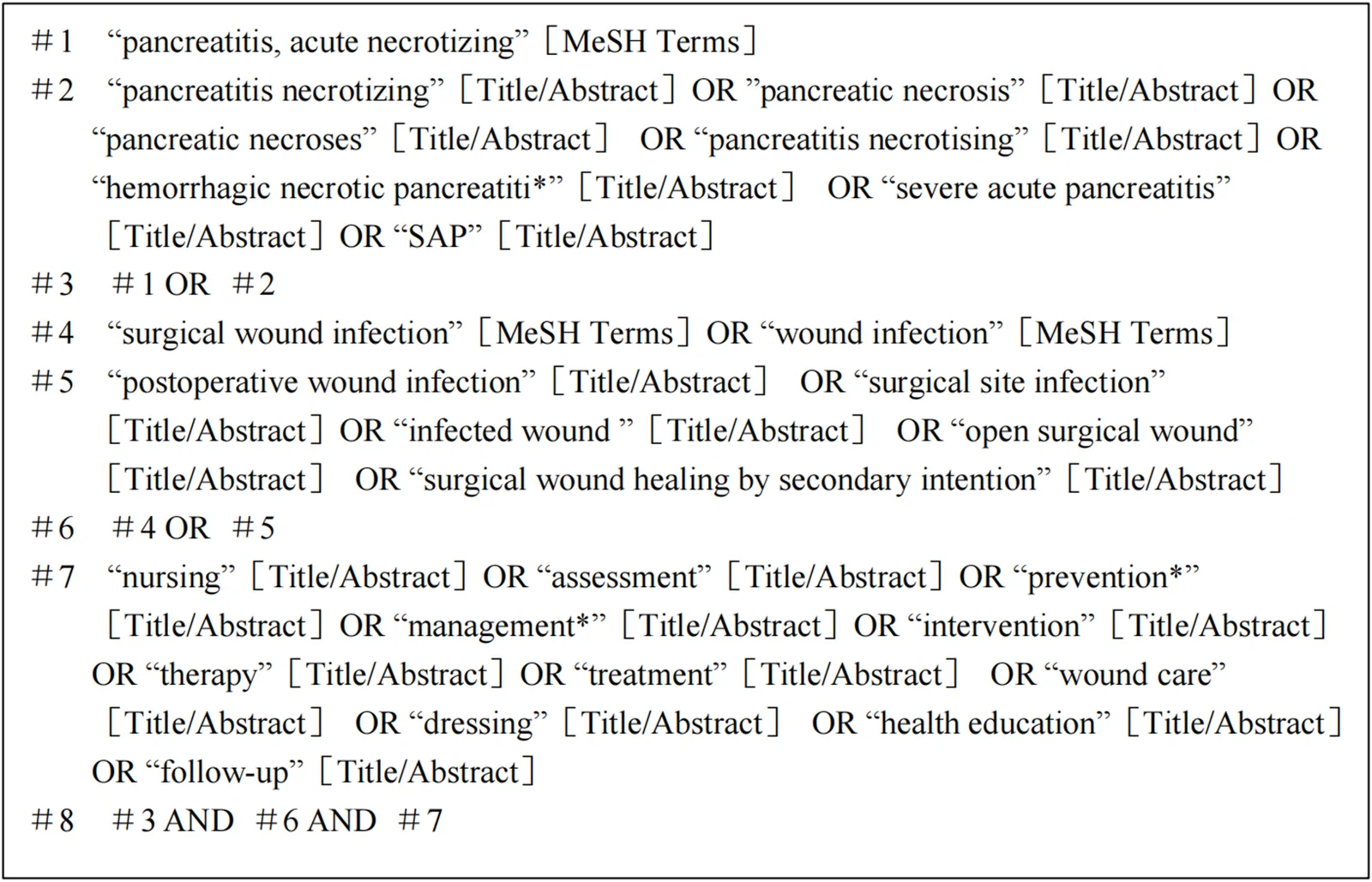
Pubmed search strategy

A comprehensive search was conducted across clinical decision-support resources, guideline platforms, wound-care professional organisations and Chinese and English bibliographic databases. These sources included UpToDate, BMJ Best Practice, the Guidelines International Network, the National Institute for Health and Care Excellence, the Scottish Intercollegiate Guidelines Network, the Registered Nurses’ Association of Ontario, the Medlive Guidelines Network, the International Wound Infection Institute, the Wound, Ostomy and Continence Nurses Society, the European Wound Management Association, Wounds Australia, Wounds Canada, the American Board of Wound Management, the Association for the Advancement of Wound Care, China National Knowledge Infrastructure, Wanfang Data, VIP, China Biology Medicine disc, PubMed, Ovid MEDLINE, Embase, CINAHL, the Cochrane Library and Web of Science. Eligible evidence sources were restricted to those published from database inception to 30 September 2025, and the PubMed strategy is shown in Fig. 1.

### Inclusion and exclusion criteria

Inclusion Criteria: The target population consisted of adult patients aged 18 years or older with severe acute pancreatitis who underwent minimally invasive surgical or drainage-based procedures and developed, or were at risk of developing, drainage-associated open surgical wounds. The content of the literature focused on nursing assessment, infection prevention and recognition, wound care, exudate management, periwound skin protection, dressing selection and change, drainage tube exit-site care, health education, follow-up or referral. The eligible literature types included clinical practice guidelines, national standards, expert consensus statements, evidence summaries, clinical decisions, best practice recommendations, practice-oriented clinical frameworks, clinical pathway or practice tools, and systematic reviews or meta-analyses. The literature was available in English or Chinese. No lower publication-date limit was imposed; evidence sources published or updated from database inception to 30 September 2025 were eligible for inclusion.

For systematic reviews and meta-analyses, eligibility was additionally assessed using the PICO framework. The population included adults with severe acute pancreatitis undergoing minimally invasive surgical or drainage-based treatment and, where condition-specific systematic reviews were unavailable, adults with open surgical wounds, wounds healing by secondary intention, drain-related wounds or comparable surgical wound complications. Eligible interventions included wound assessment, infection prevention or management, wound cleansing, debridement, exudate control, periwound skin protection, dressing selection, drainage tube exit-site care, nutritional support, negative pressure wound therapy, health education or follow-up. Comparators included usual care, no specific intervention or an alternative wound-care intervention, where applicable. Outcomes included wound infection, exudate or leakage control, periwound skin integrity, pain, wound healing and care-related burden. Systematic reviews involving broader surgical populations were included only when their findings were clinically relevant to a predefined component of the target wound-care pathway; such findings were treated as indirect evidence during evidence synthesis.

Exclusion Criteria: (1) full texts were unavailable; (2) the literature failed to meet the predefined methodological quality appraisal criteria; (3) duplicate publications or superseded versions were identified;

### Literature quality evaluation criteria

Different instruments were selected according to the type of evidence source. Clinical practice guidelines were reviewed with the Appraisal of Guidelines for Research and Evaluation II (AGREE II) tool [17], which examines guideline quality across six domains: scope and purpose, stakeholder involvement, rigour of development, clarity of presentation, applicability and editorial independence. Items are scored from 1 to 7, and domain scores are converted into standardised percentages using the AGREE II scoring method. Higher domain scores indicate stronger methodological quality and more transparent reporting. The overall guideline quality and the overall recommendation for use were also assessed. Guidelines recommended for use, either without or with modifications, were retained; guidelines not recommended for use were excluded. The A Measurement Tool to Assess Systematic Reviews 2 (AMSTAR 2) was used for systematic reviews and meta-analyses, focusing on methodological rigour, risk of bias assessment, and reporting transparency, in accordance with the recommended critical appraisal approach for systematic reviews [18]. Overall confidence in the results of each review was classified as high, moderate, low or critically low according to the number and nature of critical and non-critical weaknesses. Reviews rated as high or moderate confidence were retained, whereas those rated as low or critically low confidence were excluded from evidence extraction.

The national health industry standard, best-practice recommendation documents, international consensus document, practice-oriented clinical framework and local clinical pathway/practice tool were evaluated using the JBI Critical Appraisal Checklist for Text and Opinion Papers [19]. Each item was rated as “Yes”, “No”, “Unclear” or “Not applicable”, followed by an overall appraisal decision of “Include”, “Exclude” or “Seek further information”. Sources receiving an overall decision of “Include” were retained. Sources requiring further information were re-examined, and the final decision was reached through reviewer discussion.

### Process of literature quality evaluation

Four reviewers with evidence-based practice training assessed the eligible evidence sources independently using the appraisal tools described in Sect.  2.4. Before the main appraisal, sample documents were reviewed to calibrate judgement standards. Assessment results were then cross-checked, and differences were discussed until agreement was reached. Persistent disagreements were referred to a fifth reviewer with expertise in evidence-based nursing and wound care. For guidelines, consistency among reviewers was tested with the intraclass correlation coefficient, and values greater than 0.75 were considered acceptable. In cases of inconsistent recommendations, evidence with stronger methodological quality, higher evidence level, more recent publication, greater directness to the target wound-care context and better feasibility and safety was prioritized. Final decisions were confirmed by the multidisciplinary evidence-based practice team.

### Evidence classification, directness and recommendation level judgement

Evidence grading and recommendation strength were determined using the JBI evidence level and recommendation grading approach [20]. Two reviewers independently extracted clinically actionable recommendations from the included sources, and a third reviewer checked the extracted information against the original documents. Similar recommendations were integrated, overlapping content was removed, and the language was adjusted to reflect the clinical context of drainage-associated open surgical wounds after minimally invasive treatment for severe acute pancreatitis.

The level of evidence was assigned according to the design and nature of the supporting source, from Level 1 to Level 5, with Level 1 representing the strongest evidence and Level 5 mainly representing expert opinion or laboratory-based evidence [20]. Recommendation strength was classified as Grade A or Grade B by considering methodological quality, evidence level, consistency, clinical relevance, feasibility, appropriateness, meaningfulness, effectiveness, patient preferences and safety. The FAME framework informed the assessment of feasibility, appropriateness, meaningfulness and effectiveness [21]. Where recommendations were inconsistent, evidence with stronger quality, greater recency, closer clinical relevance and better feasibility was prioritized. Final evidence items and recommendation grades were confirmed through multidisciplinary expert discussion.

Directness was assessed separately from evidence level and recommendation strength by comparing the population, wound context, intervention and outcomes of each supporting source with the target clinical question. Evidence specifically addressing adults with severe acute pancreatitis and drainage-associated open surgical wounds following minimally invasive surgical or drainage-based treatment was considered direct. Evidence derived from general surgical wounds, closed surgical incisions, wounds healing by secondary intention, surgical-site infection prevention, moisture-associated skin damage or general wound-care practice was considered indirect [22]. The wording of each statement was calibrated to its directness and source basis: definitive wording was retained only for explicit safety standards or clearly graded recommendations, whereas indirect, low-level or practice-oriented evidence was expressed using cautious terms such as “may be considered”, “may support” or “can inform”.

### Development of the nursing decision pathway and implementation tools

After evidence extraction, classification and recommendation grading, the final evidence items were translated into a nursing decision pathway and practical implementation tools. First, the research team identified key decision points in the nursing management of drainage-associated open surgical wounds following minimally invasive treatment for severe acute pancreatitis, including risk stratification, wound type identification, infection recognition, exudate assessment, periwound skin protection, dressing selection, drainage tube exit-site care, reassessment, referral and post-discharge follow-up. Second, the evidence items were mapped to these decision points to ensure consistency between the pathway and the evidence summary. Third, evidence items requiring repeated assessment, rapid clinical judgement or procedural standardisation were converted into practical tools, including a wound assessment record form, an infection grading and management card, a dressing selection matrix, a drainage tube exit-site care checklist and a health education and follow-up plan. Finally, the draft pathway and tools were reviewed by a multidisciplinary expert panel, and revisions were made according to clinical relevance, feasibility, clarity, safety and applicability to routine nursing practice. The final pathway and tools were confirmed through group discussion.

## Results

### Literature screening process and characteristics of included studies

**Fig. 2:**
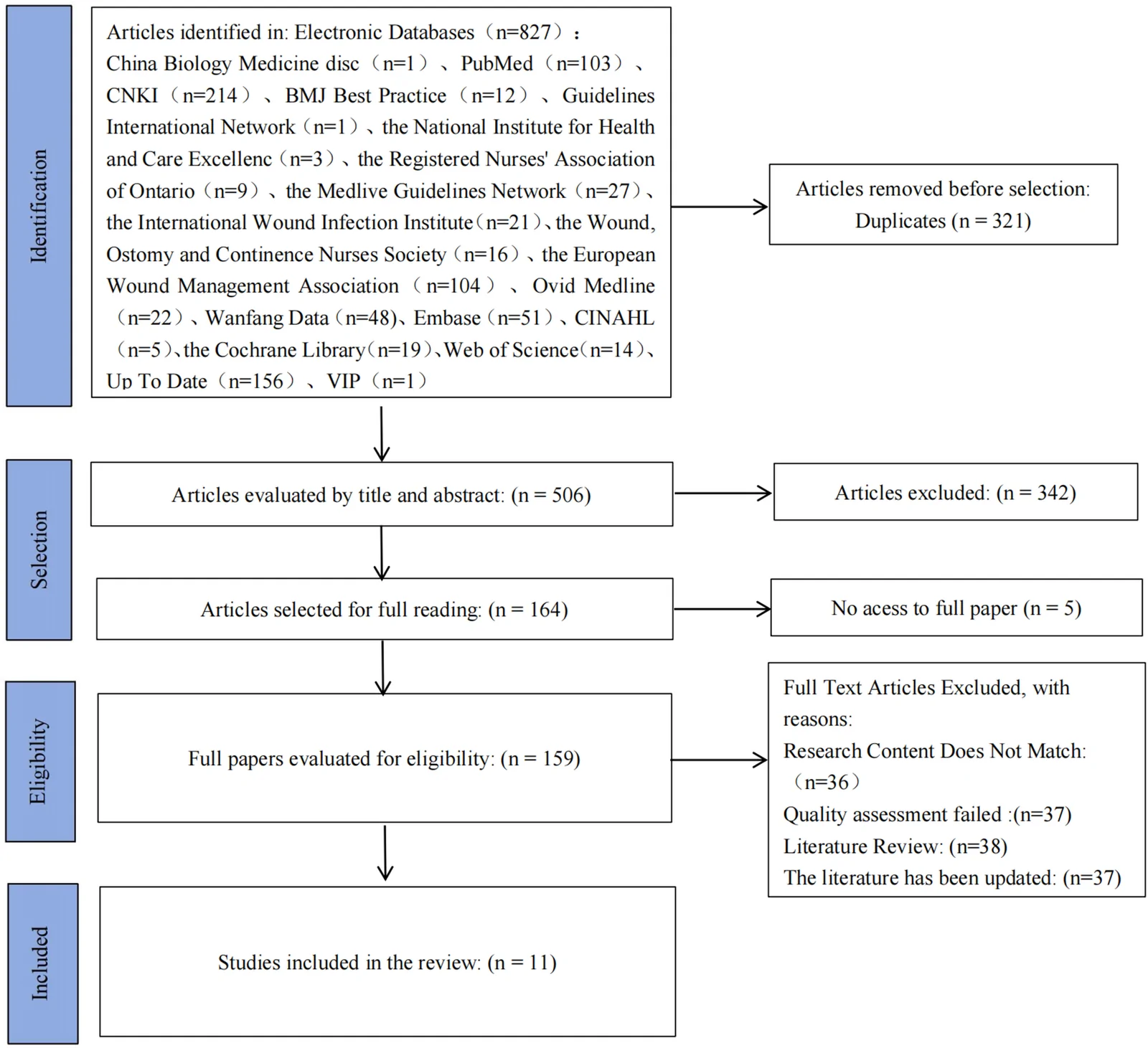
The flow chart of literature screening

In this study, a total of 827 records were retrieved from electronic databases, guideline and professional organisation websites, and clinical decision-support resources. After duplicate removal and screening of titles, abstracts and full texts, 11 evidence sources were finally included in the final synthesis. These comprised two clinical practice guidelines [6, 23], one national health industry standard [24], two best-practice recommendation documents [5, 25], three systematic reviews or meta-analyses [26–28], one international consensus document [12], one practice-oriented clinical framework [29] and one local clinical practice pathway/practice tool [30]. The literature screening process is presented in Fig. 2, and the general characteristics of the included evidence sources are shown in Table 1.

**Table 1:** Basic characteristics of the included literature (*n* = 11)

Author or issuing orgaation	Publication Year /latest update	Retrieval source	The literature theme	Type of literature
Association for the Advancement of Wound Care [23]	2018	AAWC website	Major Recommendations for the International Consolidated Wound Infection Guideline (ICWIG)	Clinical practice guideline
Harris et al. [25]	2017	Wounds Canada	Best Practice Recommendations for the Prevention and Management of Surgical Wound Complications	Best-practice recommendations
National Institute for Health and Care Excellence [6]	2019, updated 2020	NICE website	Surgical site infections: prevention and treatment	Clinical practice guideline
National Health Commission of the People’s Republic of China [24]	2025	National Health Commission official website	Standards for Prevention and Control of Surgical Site Infection	National health industry standard
Zhang et al. [26]	2025	PubMed	Influences of enteral nutritional enriched with n-3 polyunsaturated fatty acids on postoperative wound infection following gastrointestinal surgery: A meta-analysis	Systematic review and meta-analysis
Lenet et al. [27]	2022	PubMed	The impact of prophylactic negative pressure wound therapy on surgical site infections in pancreatic resection: A systematic review and meta-analysis	Systematic review and meta-analysis
Jiang et al. [28]	2020	PubMed	Evaluation of different surgical dressings in reducing postoperative surgical site infection of a closed wound: A network meta-analysis	Systematic review and network meta-analysis
Hunt and Azad [29]	2023	PubMed	ABCDEFGHI Systematic Approach to Wound Assessment and Management	Practice-oriented clinical framework
International Wound Infection Institute [12]	2022	Wounds International website	International Wound Infection Institute.Wound Infection In Clinical Practice	International consensus document
Fletcher et al. [5]	2020	Wounds International	International Best Practice Recommendations: Prevention and Management of Moisture-Associated Skin Damage (MASD)	Best Practice Recommendations
Doncaster and Bassetlaw Teaching Hospitals NHS Foundation Trust, Skin Integrity Team [30]	2025	DBTH website	Pathway for Healing by Secondary Intention Surgical Wounds	Local clinical pathway/practice tool

### Results of literature quality evaluation

#### The evaluation results of guidelines quality

The quality appraisal results for the two included clinical practice guidelines are presented in Table 2. The ICC values for AGREE II appraisal ranged from 0.835 to 0.982, indicating good consistency among reviewers. Both guidelines were rated as Grade A and one as Grade B.

#### The evaluation results of systematic reviews quality

The quality appraisal results for the three included systematic reviews or meta-analyses are presented in Table 3.

#### The evaluation results of text- and opinion-based evidence sources

The JBI appraisal results for four text- and opinion-based evidence sources—the national health industry standard, one best-practice recommendation document, the practice-oriented clinical framework and the international consensus document are presented in Table 4.

#### The evaluation results of additional best-practice and pathway documents

The quality appraisal results for the additional best-practice recommendation document and local clinical pathway/practice tool are presented in Table 5.

**Table 2:** Quality evaluation results of the included clinical practice guidelines (*n* = 2)

Included studies	Normalised percentage in each area (%)								Overall evaluation		
	Scope and purpose	Relevant personnel participation	Rigour of development	Clarity of guidance	Applicability of the guideline	Independence of the guideline	Number of areas ≥ 60%	Number of areas ≥ 30%	Overall quality score of the guideline (score)	Is this guideline recommended?	Recommendation level
AAWC [23]	89	85	86	92	78	90	6	6	5.25	Recommended	B
NICE [6]	100	92	96	93	94	92	6	6	6.75	Recommended	A

**Table 3:** Quality evaluation results of the included systematic reviews and meta-analyses (*n* = 3)

Evaluation items	Zhang [26]	Lenet [27]	Jiang [28]
1.Did the research questions and inclusion criteria for the review include the components of PICO?	Yes	Yes	Yes
2.Did the report of the review contain an explicit statement that the review methods were established prior to the conduct of the review and did the report justify any significant deviations from the protocol?	Partial Yes	Yes	Partial Yes
3.Did the review authors explain their selection of the study designs for inclusion in the review?	Yes	Yes	Yes
4.Did the review authors use a comprehensive literature search strategy?	Yes	Yes	Yes
5.Did the review authors perform study selection in duplicate?	Yes	Yes	No
6.Did the review authors perform data extraction in duplicate?	Yes	Yes	No
7.Did the review authors provide a list of excluded studies and justify the exclusions?	No	No	Partial Yes
8.Did the review authors describe the included studies in adequate detail?	Yes	Yes	Yes
9.Did the review authors use a satisfactory technique for assessing the risk of bias (RoB) in individual studies that were included in the review?	Yes	Yes	Yes
10.Did the review authors report on the sources of funding for the studies included in the review?	No	Yes	No
11.If meta-analysis was performed, did the review authors use appropriate methods for statistical combination of results?	Yes	Yes	Yes
12.If meta-analysis was performed, did the review authors assess the potential impact of RoB in individual studies on the results of the meta-analysis or other evidence synthesis?	Partial Yes	Yes	No
13.Did the review authors account for RoB in individual studies when interpreting/discussing the results of the review?	Partial Yes	Yes	Partial Yes
14.Did the review authors provide a satisfactory explanation for, and discussion of, any heterogeneity observed in the results of the review?	Yes	Partial Yes	Partial Yes
15.If they performed quantitative synthesis, did the review authors carry out an adequate investigation of publication bias (small study bias) and discuss its likely impact on the results of the review?	Partial Yes	No	Partial Yes
16.Did the review authors report any potential sources of conflict of interest, including any funding they received for conducting the review?	No	No	No
Overall quality rating	Moderate	High	Moderate

**Table 4:** JBI critical appraisal results for text- and opinion-based evidence sources (*n* = 4)

Evaluation items	National standard [25]	Harris [24]	Hunt and Azad [29]	IWII [12]
1. Is the source of the opinion clearly identified?	Yes	Yes	Yes	Yes
2. Does the source have standing in the field of expertise?	Yes	Yes	Yes	Yes
3. Are the interests of the relevant population the central focus?	Yes	Yes	Yes	Yes
4. Is the stated position the result of an analytical process, and is the reasoning logical?	Unclear	Yes	Yes	Yes
5. Is there reference to the extant literature?	Yes	Yes	Yes	Yes
6. Is any incongruence with the literature or sources logically defended?	Not applicable	Unclear	No	Yes
Overall appraisal	Include	Include	Include	Include

**Table 5:** JBI critical appraisal results for the additional best-practice and pathway documents (*n* = 2)

Evaluation items	Fletcher et al. [5]	DBTH Skin Integrity Team [30]
1. Is the source of the opinion clearly identified?	Yes	Yes
2. Does the source of opinion have standing in the field of expertise?	Yes	Yes
3. Are the interests of the relevant population the central focus of the opinion?	Yes	Yes
4. Is the stated position the result of an analytical process, and is there logic in the opinion expressed?	Yes	Unclear
5. Is there reference to the extant literature?	Yes	Yes
6. Is any incongruence with the literature/sources logically defended?	Unclear	Not applicable
Overall evaluation results	Include	Include

### Best evidence synthesis

This study provides a comprehensive overview of the evidence for the nursing management of drainage-associated open surgical wounds following minimally invasive treatment for severe acute pancreatitis. It encompasses multidisciplinary teamwork, wound assessment and monitoring, systemic optimisation and infection management, cleansing and debridement exudate management and periwound skin protection, dressing selection and change, drainage tube exit-site care, health education and follow-up, and decision pathways and supporting tools. Because direct evidence specifically targeting this combined clinical and wound context was limited, several recommendations were derived from expert consensus, practice-oriented documents or evidence from broader surgical populations. Where recommendations were based on indirect or lower-level sources, cautious wording was applied in the evidence summary to reflect the nature of the supporting evidence and to avoid overgeneralisation to the target population. In total, 35 evidence items are presented in Table 6.

**Table 6:** Summary of best evidence for the nursing management of drainage-associated open surgical wounds following minimally invasive treatment for severe acute pancreatitis

Evidence category	Evidence description	Evidence Level (Grade)	Recommendation level
Teamwork	1.Wound management requires multidisciplinary team collaboration involving physicians, nurses, wound therapists, pharmacists, dietitians, and psychological counselors [12, 23, 25].	5	B
	2.Encourage patients and their caregivers to participate in wound care decision-making to reduce physiological and psychological impacts on patients [12, 23, 25].	5	B
Assessment and Monitoring of Open Surgical Wounds Related to Drainage	3.Evaluate individual factors contributing to wound infection: (1) Assess the patient’s medical history, comorbidities, medication use, nutritional status, factors influencing inflammatory and immune responses, factors affecting local tissue healing, and psychosocial support [12, 23, 25]. (2) Assess the patient’s smoking history; a smoking history within 4 weeks prior to surgery increases the risk of postoperative wound infection [23]. (3) Assess whether the patient exhibits behaviors that may expose wounds to infectious microorganisms [23]. (4) Evaluate the learning styles of the patient and caregivers, their comprehension of health guidance, and their ability to participate in wound infection prevention measures [23].	4	B
	4.Assess environmental factors contributing to wound infection: including microbial load on air and surfaces in the patient’s hospital room, compliance with healthcare worker hand hygiene practices, and adherence to aseptic technique implementation protocols [12].	5	B
	5.Local wound assessment: (1)Postoperative wound assessments should be performed daily, with the frequency determined by the type of wound dressing used [12]. (2) Comprehensive, standardized, and applicable wound infection risk assessment tools are recommended, although no single tool can provide a complete evaluation of patients, wounds and environments. Clinical judgment should still be integrated to inform wound assessment decisions [12]. (3) Wound infection assessments should be conducted by multidisciplinary wound management team members with specialized training, and results should be communicated to relevant patient-care personnel [23]. (4) Assessment criteria include wound color, periwound oedema and erythema, induration, fever, pain, exudate volume, exudate characteristics, odor, and granulation tissue formation [25, 29].	5	B
	6.Wound infection should be identified based on wound symptoms and patient signs, but it is important to note that immunocompromised individuals may not exhibit typical signs of infection. The color of wound edges and induration cannot serve as reliable indicators of wound infection, as these characteristics may vary due to individual differences in skin pigmentation [12].	5	B
	7.Microbiological testing should be performed when there is significant spread of infection at the wound site, or when the wound worsens, enlarges, or shows poor healing despite anti-infective treatment. However, culture results should not be used as an independent basis for diagnosing wound infection [23], Samples can be collected from wounds through tissue biopsy, wound fluid aspiration, sharp debridement, and wound swabs. Tissue biopsy is the preferred method for obtaining accurate cultures, as it provides both quantitative and qualitative information. The Levine technique for collecting wound swabs is the most commonly used in clinical practice, facilitating the removal of microorganisms beneath the wound tissue. Prior to sample collection, the wound should be irrigated with normal saline to remove visible necrotic or decaying tissue, followed by drying with sterile gauze. Disinfectants should not be used [12].	5	B
	8.Professional healthcare providers evaluate whether wound care meets the intended objectives using standardized tools and methods, combined with patient feedback. If partial achievement or failure to meet the objectives is identified, the patient, wound, environment, and support systems are reassessed, and the care plan is adjusted accordingly [25].	5	B
Infection Prevention and Antimicrobial Strategies	9.Patients are advised to cleanse their skin one day prior to the surgical procedure [6, 23, 24]. However, the use of antibacterial disinfectants for cleaning is not recommended [23].	3	A
	10.Through effective air filtration or treatment and surface sterilization techniques, monitor and minimize microbial loads in the air and surfaces of operating rooms and patient wards, thereby reducing personnel movement within these areas [23].	1	A
	11.Intraoperative thermoregulatory measures were administered to the patient [6, 23, 24], maintain core body temperature ≥ 36.0 °C [24].Surgical irrigation fluid, blood transfusion, and intravenous fluids should be warmed to approximately 37.0 °C, and water bath heating is not recommended [23].	1	A
	12.Preoperative assessment of the patient’s blood glucose level is essential. Regardless of whether the patient has diabetes mellitus, perioperative blood glucose control should be maintained. The attending physician should comprehensively determine the target blood glucose control level for the patient [6, 23, 24].	1	A
	13.Assess the patient’s oxygenation index preoperatively, and immediately increase the inhaled oxygen concentration (FiO2) to 0.3–0.4 during or immediately after extubation [24], to ensure that the patient’s blood oxygen saturation remains above 95% [12, 23].	1	A
	14.Evidence from gastrointestinal surgery suggests that enteral nutrition supplemented with n-3 polyunsaturated fatty acids may reduce postoperative wound infection. Although direct evidence in patients with drainage-associated open wounds secondary to severe acute pancreatitis is lacking, these findings may provide indirect supportive evidence for nutritional management and should be interpreted with caution [26].	5	B
	15.Routine prophylactic negative pressure wound therapy (NPWT) should not be assumed to be beneficial for all high-risk surgical wounds. For open surgical wounds healing by secondary intention or drainage-associated wounds, NPWT should be considered only after assessment of wound characteristics, patient burden, local expertise, cost and available resources [27].	1	A
	16.For wounds with suspected or confirmed biofilm formation or local infection, topical antimicrobial therapy may be considered as part of a structured infection-management plan, followed by reassessment after approximately 2 weeks. If spreading infection or systemic infection is suspected, topical antimicrobial therapy should not be used alone and should be combined with medical review and systemic antimicrobial treatment when clinically indicated [12].	5	B
Exudate Management and Peri-dermal Protection (MASD Prevention and Control)	17.Wound cleaning should commence during dressing changes within 48 h postoperatively, followed by routine wound cleansing at each subsequent dressing change. Care must be taken to thoroughly remove contaminants, debris, necrotic tissue, and exudates from the wound surface to promote healing [6, 12].The use of wet gauze for wound cleaning is not recommended as an alternative to proper wound hygiene [12].	5	B
	18.For wounds with infection or extensive necrotic tissue, rigorous therapeutic debridement of the wound surface is required. ① Administer analgesics as prescribed by the physician prior to therapeutic debridement. ② During debridement, use an adequate volume of solution (approximately 50–100 mL per centimeter of wound length), maintain irrigation pressure at 4–15 psi. ③ The debridement area should encompass the entire wound dressing coverage or 10–20 cm of surrounding skin beyond the wound edges [12].	5	B
	19.It is recommended to routinely clean wounds with sterile normal saline [12, 25], and surfactants may also be used to distribute body fluids uniformly over the wound bed while removing bacteria without damaging healing tissue, thereby providing a stable moist environment for wound healing [12]. For cases of localized or spreading wound infections, disinfectants such as peroxyacetic acid solution or povidone-iodine should be used with caution [12].	5	B
	20.It is recommended to use a solution at room temperature or slightly warm temperature when cleaning wounds [12].	5	B
	21.When there is significant exudate from drainage-associated open surgical wounds or prolonged irritation by drainage fluid, periwound skin protection should be established as a core nursing objective. Barrier films, liquid polymers or acrylic terpolymer barrier products may be used within the dressing or fixation area to form a protective layer that reduces exposure to moisture and irritants and lowers the risk of maceration [5].	5	B
	22.When there is a risk of periwound skin maceration, erythema, erosion or medical adhesive-related skin injury (MARSI) caused by frequent adhesive removal, low-allergenic, easily removable silicone-based dressings or fixation methods should be prioritized. The effect of barrier products on dressing adhesion and absorption should be evaluated and products should be used according to manufacturer instructions [5].	5	B
Wound dressing selection and replacement	23.Postoperatively, use a moistening closed dressing to protect the wound, and replacement should be performed within 24–48 h [23, 25].	1	A
	24.When changing wound dressings, the procedure should be performed by trained healthcare professionals using sterile non-contact techniques for removal and replacement of the dressing [6, 23, 24].	1	A
	25.The selection of appropriate dressings should be based on wound changes and exudate volume, combined with input from wound therapists, physicians, patients, and caregivers, while comprehensively considering infection risks and cost factors [6, 23, 25]. It is recommended to use hydrocolloids and film dressings for minor exudation, hydrogel fibers or alginate dressings for mild to moderate exudation, and foam dressings for significant exudation [29].	5	B
	26.Evidence from closed postoperative wounds suggests that some advanced or antimicrobial dressings, such as mupirocin-containing dressings, dialkylcarbamoyl chloride dressings and vitamin E-silicone dressings, may reduce postoperative surgical site infection in selected settings. However, this evidence is indirect for drainage-associated open surgical wounds after severe acute pancreatitis; therefore, these dressings should not be assumed to be beneficial for all patients and should be selected according to wound type, infection risk, exudate level, periwound skin condition, patient preference, cost and local availability [28]	1	A
	27.When clinical signs of local wound infection are present, antimicrobial dressings may be selected according to wound condition, exudate level, periwound skin tolerance, dressing availability and local protocols. Options may include silver-containing dressings, chlorhexidine dressings, polyhexamethylene biguanide dressings, methylene blue/gentian violet dressings or iodine-containing dressings. Because this evidence is largely derived from broader wound-care contexts rather than drainage-associated open surgical wounds after severe acute pancreatitis, dressing selection should be individualized and regularly reassessed [29].	5	B
Management of Drainage Tubes	28.For leakage associated with double-lumen or large-bore drainage systems, a combined strategy of leakage isolation, drain fixation and periwound skin protection can be adopted. Drainage tubes should be securely fixed to minimize traction. when necessary, drainage collection bags, ostomy appliances or other isolation devices may be used under the guidance of wound therapists to reduce skin exposure to wound fluid [5, 25].	5	B
Health Education and Follow-up	29.Health education should be conducted based on the individualized needs of patients and their caregivers [25]. The content should include: members and responsibilities of multidisciplinary wound management teams [6, 25]; risks and preventive measures for surgical site infections [6, 23, 25]; symptoms and signs of surgical site infections [6, 23, 25]; principles of hand hygiene, wound care knowledge, use of wound dressings, and how to identify and address wound issues [25].	5	B
	30.Health education should cover all stages of wound management [23, 25], with appropriate methods selected based on the age and educational level of patients and their caregivers [23]. These methods include face-to-face guidance, instructional materials, and digital tools [25].	4	B
	31.Provide preoperative guidance to patients including smoking cessation, blood glucose control, and nutritional counseling [25].	5	B
	32.Provide patients with psychosocial support [12, 25].	5	B
	33.Secondary wound healing after open surgery should be systematically reassessed during each dressing change, with treatment regimens reviewed at least every 2 weeks. If no improvement is observed for 14 consecutive days or if the case is complex and challenging (deep cavity, persistent massive exudation, or failed infection control), referral to a wound specialty team or tissue repair team should be considered [30].	5	B
	34.Post-discharge extended nursing care can be combined with mobile health follow-ups (such as WeChat mini-programs/remote photo assessment) and stratified follow-up reminders to enhance patients’ self-management capabilities and early infection detection [25].	5	B
Decision -making Path and Conversion Tools	35.The IWII wound infection continuum (contamination/colonization/local infection/spreading infection/systemic infection) and the T.I.M.E.S wound bed preparation framework can be integrated into the nursing decision pathway to guide debridement, cleansing, antimicrobial dressing selection, escalation and re-evaluation nodes [12, 30].	5	B

### Decision pathway and translation tools

Based on the 35 evidence items, the research team developed a nursing decision pathway for drainage-associated open surgical wounds following minimally invasive treatment for severe acute pancreatitis. The pathway was structured around eight key decision nodes: risk stratification, wound type identification, infection recognition, exudate and periwound skin assessment, dressing and antimicrobial strategy selection, drainage tube exit-site care, reassessment and referral, and post-discharge follow-up. These nodes were mapped to the corresponding evidence items and integrated with the IWII wound infection continuum, secondary intention wound reassessment principles and best-practice recommendations for periwound skin protection and moisture-associated skin damage prevention [5, 12, 30]. The pathway should be interpreted as an evidence-informed decision-support tool that requires local adaptation and clinical validation rather than as a fully validated clinical algorithm. The nursing decision pathway is presented in Table 7.

To support pathway implementation, five practical tools were developed: a drainage-associated open surgical wound assessment record form, an infection grading and management card, a dressing selection matrix, a drainage tube exit-site care checklist and a health education and follow-up plan. These tools correspond to the major decision nodes in the pathway and are intended to support repeated assessment, infection staging, dressing selection, drainage tube exit-site care, discharge education and follow-up management.

**Table 7:** Nursing decision pathway for the nursing management of drainage-associated open surgical wounds following minimally invasive treatment for severe acute pancreatitis

Decision node	Key assessment points (operable)	Recommended actions/tools (corresponding evidence)
A. Risk stratification	Assess for malnutrition/high blood sugar/ immunosuppression, continuous lavage, frictional traction of drainage tubes, and prior SSI (Surgical Site Infection).	T1 ‘Drainage-Related Open Surgical Wound Assessment Record Form, based on Evidence 3–5.
B. Wound type identification	Incompatible incision edges, persistent exudation, or drainage-related moist environment → secondary healing of open wounds.	Set re-evaluation nodes according to the second-phase healing pathway; Evidence 33.
C. Infection recognition	Identify local, spreading or systemic infection using the IWII wound infection continuum; collect microbiological samples when clinically indicated.	T2 Infection Classification and Management Card; Evidence 6–7, 35.
D. Exudation and MASD Prevention and Control	Assess exudate volume and characteristics, odor, periwound maceration/erosion, barrier-product need and adhesive injury risk.	Barrier products + absorbent dressings + low-trauma fixation; Evidence 21–22, 25–28.
E. Wound dressing and antimicrobial strategies	Select antimicrobial, absorbent or packing dressings according to exudate volume, wound depth, infection severity and periwound skin tolerance; avoid unnecessary or excessive irrigation.	T3 Wound Dressing Selection Matrix; Evidence 23, 25–27, 35.
F. Drainage tube exit-site care	Assess for leakage, traction, fixation point pressure, and skin lacerations.	T4 Drainage Tube Exit-Site Care Checklist; Evidence 17–20, 28.
G. Re-evaluation and Referral	Re-evaluation after each dressing change; no improvement after ≥ 14 days or failure of complex deep cavity/infection control.	Hierarchical referral to wound specialty; Evidence 33.
H. Post-discharge follow-up	Assess patients’ learning ability and family care resources.	T5 Health Education Key Points Card + Follow-up Plan; Evidence 29–32, 34.

## Discussion

### Establish multidisciplinary collaboration and patient participation in wound management

Evidence items 1–2 indicate that drainage-associated open surgical wound management should be supported by multidisciplinary collaboration and patient participation. Patients recovering from minimally invasive treatment for severe acute pancreatitis often require simultaneous infection control, drainage tube management, wound assessment, nutritional support, pain management and psychological support [1, 12]. Therefore, wound care should not be regarded as the responsibility of nurses alone. Current surgical wound and wound infection guidance also emphasises the role of coordinated decision-making and patient engagement in wound management [12, 25]. In clinical practice, hepatobiliary and pancreatic surgeons, specialist nurses, wound/ostomy therapists, infection-control practitioners, pharmacists, dietitians and psychological counsellors should clarify their respective responsibilities. Nurses may act as the coordinator of ongoing wound assessment, dressing change planning, patient education and referral communication. Patients and caregivers should be involved in decisions on dressing tolerance, pain reporting, discharge preparation and follow-up arrangements, and their understanding of warning signs should be checked before discharge.

### Conduct dynamic risk assessment and standardised wound monitoring

Evidence items 3–8 describe the assessment of patient-related, environment-related and local wound-related factors. The purpose of assessment is to identify high-risk patients, detect early wound deterioration and determine whether the wound care plan should be adjusted [12, 29]. Drainage-associated open wounds differ from closed incisions because persistent drainage, lavage-fluid leakage, tube traction, repeated dressing changes and periwound irritation may change local wound appearance and make infection recognition more difficult [4, 5, 12]. The IWII wound infection continuum provides a practical framework for identifying contamination, colonisation, local infection, spreading infection and systemic infection, but the diagnosis of wound infection should still be based on combined clinical judgement rather than culture results alone [12]. Nurses should complete a baseline assessment after surgery and repeat assessment at each dressing change, including medical history, comorbidities, nutrition, immune status, smoking history, hygiene practices, learning ability, wound size and depth, wound bed, exudate amount and character, odour, pain score, periwound erythema or maceration, drainage tube fixation, leakage and systemic symptoms. If pain increases, exudate becomes purulent or malodorous, erythema spreads, fever develops, the wound enlarges or healing is delayed, medical review, wound specialist consultation or microbiological sampling should be initiated according to local protocols.

### Combine systemic optimisation with infection management

Evidence items 9–16 address blood-glucose management, nutritional support, prophylactic negative pressure wound therapy and antimicrobial management. These issues are relevant to severe acute pancreatitis because systemic inflammation, stress hyperglycaemia, nutritional compromise, prolonged hospitalisation and ongoing intra-abdominal infection may affect wound repair and infection risk [1, 6]. However, the available evidence does not directly address drainage-associated open wounds in severe acute pancreatitis. The meta-analysis of n-3 polyunsaturated fatty acid-enriched enteral nutrition was conducted in broader gastrointestinal surgical populations [26], while the negative-pressure wound therapy review concerned prophylactic treatment of primarily closed pancreatectomy incisions [27]. These findings may inform selected decisions but should not be extrapolated as routine treatment requirements for an established open wound. Nurses should monitor temperature, pain, blood glucose where relevant, nutritional risk, drain output, wound exudate and signs of local, spreading or systemic infection. Nutritional formulations and systemic antimicrobial therapy should be determined through multidisciplinary assessment, and topical antimicrobial treatment should be time-limited and reviewed for clinical response, especially when direct evidence for their efficacy in this specific wound type remains limited [12].

### Manage exudate, periwound skin, dressing selection and drainage tubes as an integrated bundle

Evidence items 17–28 focus on wound cleansing, debridement, exudate control, periwound protection, dressing selection, dressing change and drainage tube-related management. For drainage-associated open surgical wounds, exudate is not only an indicator of wound status but also a major contributor to periwound maceration, dressing failure, odour, pain, adhesive injury and increased nursing workload [5, 12]. International best practice recommendations for moisture-associated skin damage highlight the need for skin-focused prevention and management plans in patients exposed to moisture or irritant fluids [5]. Dressing selection should therefore be based on exudate volume, infection status, wound depth, cavity or undermining, periwound skin tolerance, pain and dressing-change frequency. Film or hydrocolloid dressings may be considered for minimal exudate, hydrofiber or alginate dressings for low-to-moderate exudate, and foam or other absorbent dressings for heavier exudate, while antimicrobial dressings should be used according to local infection signs and reassessed regularly. Evidence suggesting benefit from particular dressings was derived from closed surgical incisions [28] and should not be interpreted as establishing a preferred product for drainage-associated open wounds. Similarly, evidence on negative pressure wound therapy for wounds healing by secondary intention remains primarily derived from broader surgical populations and should not be adopted as routine without careful consideration of wound characteristics, patient burden and local resources [29]. In clinical practice, nurses should inspect exudate volume, colour, viscosity and odour; assess periwound maceration, erythema, erosion, pain and adhesive-related injury; check drainage tube patency, fixation, traction, leakage and fixation-point pressure; and use barrier products, low-trauma fixation, absorbent dressings or collection/isolation devices when persistent leakage or periwound damage is present.

### Extend wound care through individualised education and follow-up

Evidence items 29–34 indicate that health education and follow-up should be integrated throughout the wound management process. Drainage-associated open surgical wounds may require prolonged care after discharge, and patients or caregivers may need to observe wound changes, protect the drainage exit site, manage dressings and recognise signs requiring medical attention. NICE recommends that patients and carers receive information on wound care after discharge, how to recognise surgical site infection and who to contact if they have concerns [6]. Education should therefore include hand hygiene, wound protection, drain-related precautions, dressing care, signs of local and systemic infection, pain reporting, nutrition, activity precautions and referral pathways. Before discharge, nurses should assess the patient’s age, health literacy, psychological status, family support and home-care capacity and confirm that the patient or caregiver can identify fever, increased pain, spreading erythema, malodour, sudden increase in exudate, tube dislodgement or uncontrolled leakage. Follow-up plans should specify review timing, escalation criteria and urgent contact pathways; the mode of follow-up should be adapted to clinical risk and local resources.

### Use decision pathways and implementation tools to support evidence translation

Evidence item 35 suggests that the IWII wound infection continuum and secondary intention wound reassessment principles can be integrated into a nursing decision pathway to improve the operability of evidence in complex wound care. The originality of this study lies not in generating new primary clinical-effectiveness evidence, but in translating dispersed evidence from wound infection, surgical site infection prevention, moisture-associated skin damage, secondary intention wound care and drainage tube care into a sequential decision-support pathway for a complex clinical population. For drainage-associated open wounds after minimally invasive surgery for severe acute pancreatitis, a list of evidence items alone may be insufficient because nurses must determine the order of assessment, when to escalate care, how to select dressings and how to coordinate infection recognition, exudate control, periwound protection, drainage tube care and follow-up [29, 31]. The nursing decision pathway developed in this study links risk stratification, wound type identification, infection recognition, exudate and periwound assessment, dressing selection, drainage tube exit-site care, reassessment, referral and post-discharge follow-up. The five supporting tools were developed by the research team to operationalise evidence items 1–35 and should be regarded as implementation aids rather than additional evidence items. During future implementation, units may use audit and feedback methods to monitor documentation completeness, dressing leakage, periwound skin damage, timeliness of infection escalation, referral rate, patient education completion and follow-up adherence.

### Implications for the nursing process and professional taxonomies

The findings may also support more standardised documentation and care planning within the nursing process by providing clinically specific content that can be mapped to established nursing taxonomies, including NANDA-I nursing diagnoses, NIC-type interventions and NOC-type outcomes. These implications should not be interpreted as formal revisions to international taxonomies; rather, they indicate how the synthesised evidence may be incorporated into local nursing documentation systems, electronic health records and institutional care plans for patients with drainage-associated open surgical wounds after minimally invasive treatment for severe acute pancreatitis.

At the assessment and nursing diagnosis stages, the evidence highlights risk factors and clinical cues that may inform care plans related to Risk for Infection, Risk for Impaired Skin Integrity, Impaired Skin Integrity, Impaired Tissue Integrity, Acute Pain and Deficient Knowledge. Relevant risk factors may be grouped into three categories: patient- and systemic-level factors, such as malnutrition, hyperglycaemia, immunosuppression, smoking and limited self-care capacity; wound- and drainage-level factors, such as wound depth, wound-bed tissue type, exudate volume and characteristics, peritubal leakage, local infection signs, mechanical traction from large-bore or multi-lumen drainage catheters and periwound skin damage; and care-context factors, such as adherence to aseptic technique, caregiver support, dressing availability and access to specialist referral. Clinical cues requiring reassessment include increasing pain, malodour, purulent exudate, spreading erythema, delayed healing, tube-site leakage and periwound skin breakdown. Nursing diagnoses should be confirmed from the patient’s actual responses and assessment findings rather than assigned solely on the basis of the medical diagnosis or surgical procedure [32].

At the planning and intervention stages, the proposed pathway provides more concrete operational content for NIC-type interventions [33]. For example, Wound Care and Incision Site Care may be operationalised through structured wound-bed assessment, wound cleansing, appropriate debridement support, antimicrobial strategy selection, dressing selection according to exudate level and scheduled reassessment. Skin surveillance and infection protection may be strengthened by systematic monitoring of periwound maceration, erythema, erosion, pain, odour, purulent exudate and signs of spreading or systemic infection. Tube care may be refined through a combined approach of leakage isolation, secure drain fixation, traction reduction and periwound skin protection, particularly for large-bore or multi-lumen drainage systems. Teaching, discharge planning and follow-up may be supported by patient education on wound observation, drainage tube precautions, infection warning signs, dressing care and referral pathways.

At the evaluation stage, the pathway may also support the selection of NOC-type outcomes [34] related to skin and tissue integrity, wound healing by secondary intention, infection severity, pain control, knowledge acquisition, self-management capacity and follow-up adherence. Corresponding nursing-sensitive outcomes may include control of infection progression, containment of exudate, maintenance of periwound skin integrity, pain relief, improvement in the wound-healing trajectory and patient or caregiver self-management competence. Future implementation studies should evaluate whether embedding these pathway elements into electronic nursing records improves assessment completeness, intervention adherence, referral timeliness and patient outcomes.

### Limitations

Although this study summarised the best available evidence for drainage-associated open surgical wounds following minimally invasive treatment for severe acute pancreatitis, several limitations should be acknowledged. First, direct high-quality evidence focusing specifically on this wound type was scarce; therefore, some recommendations were adapted from broader evidence on surgical site infection, wound infection, moisture-associated skin damage, surgical wound complications and wounds healing by secondary intention. Second, several included evidence sources were guidelines, expert consensus documents and practice-oriented documents. These sources are valuable for clinical decision-making but provide lower certainty than prospective studies in the exact target population, and they cannot replace prospective studies or establish intervention effectiveness in this specific population. Third, direct and indirect evidence were interpreted through multidisciplinary discussion, which may introduce contextual judgement despite the use of standard appraisal tools. Fourth, this evidence summary included only Chinese and English literature, which may have excluded relevant evidence published in other languages. Fifth, the nursing decision pathway and implementation tools were developed through evidence synthesis and expert discussion but have not yet been prospectively tested in clinical practice. Their feasibility, acceptability and clinical utility therefore remain to be confirmed. Sixth, the proposed mapping to NANDA-I, NIC and NOC taxonomies is conceptual and has not undergone formal cross-mapping, content validation or clinical testing. Future studies should evaluate their feasibility, acceptability, inter-rater consistency, adherence, safety, cost implications and effects on wound infection, periwound skin damage, pain, healing trajectory, unplanned readmission, and nursing workload.

## Conclusion

This study synthesised 35 indirect evidence statements and translated dispersed recommendations on wound infection, secondary-intention healing, exudate management, periwound skin protection and surgical wound care into an operational nursing decision pathway and five supporting tools for drainage-associated open surgical wounds following minimally invasive treatment for severe acute pancreatitis. Its main contribution to nursing practice is the organisation of heterogeneous evidence into sequential assessment, escalation, intervention, reassessment and follow-up decisions. Because several statements were adapted from related populations and lower-level or practice-oriented sources, the pathway should be used as an evidence-informed clinical reference rather than a prescriptive or validated protocol. Local adaptation, staff preparation and prospective evaluation are required before routine implementation. Future research should evaluate its effects on wound infection, periwound skin integrity, pain, healing trajectory and nursing workload.

## Relevance to clinical practice

Drainage-associated open surgical wounds following minimally invasive treatment for severe acute pancreatitis are difficult to manage because they involve persistent exudation, leakage, periwound skin vulnerability, drainage tube traction, infection risk and prolonged healing. This study translates 35 indirect evidence statements into a nursing decision pathway and supporting tools. The pathway may help nurses conduct structured wound assessment, recognise infection progression, select appropriate dressings, protect periwound skin, standardise drainage tube exit-site care and plan follow-up. In clinical practice, risk assessment should be conducted at baseline and updated when the wound or patient condition changes; exudate, periwound skin condition and drainage tube status should be documented together; dressing selection should be adjusted according to wound status and patient tolerance; and patients and caregivers should receive clear instructions on wound care, warning signs and contact pathways. Implementation should be accompanied by local adaptation, staff training, documentation audit and outcome evaluation.

## Data Availability

No primary datasets were generated or analysed during the current study. All data supporting this evidence summary are derived from the published evidence sources cited in this article.

## Abbreviations

AGREE II: Appraisal of Guidelines for Research and Evaluation II
MASD: Moisture-Associated Skin Damage
NPWT: Negative Pressure Wound Therapy
IWII: International Wound Infection Institute
MARSI: Medical Adhesive-Related Skin Injury
NIC: Nursing Interventions Classification
PICO: Population, Intervention, Comparator and Outcomes
PIPOST: Population, Intervention, Professional, Outcome, Setting and Type of evidence
